# Solvothermal-Based Lignin Fractionation From Corn Stover: Process Optimization and Product Characteristics

**DOI:** 10.3389/fchem.2021.697237

**Published:** 2021-08-05

**Authors:** Punjarat Khongchamnan, Wanwitoo Wanmolee, Navadol Laosiripojana, Verawat Champreda, Nopparat Suriyachai, Torpong Kreetachat, Chainarong Sakulthaew, Chanat Chokejaroenrat, Saksit Imman

**Affiliations:** ^1^ School of Energy and Environment, University of Phayao, Muang Phayao, Thailand; ^2^ National Nanotechnology Center, National Science and Technology Development Agency, Thailand Science Park, Pathumthani, Thailand; ^3^ The Joint Graduate School for Energy and Environment (JGSEE), King Mongkut’s University of Technology Thonburi, Bangkok, Thailand; ^4^ BIOTEC–JGSEE Integrative Biorefinery Laboratory, National Center for Genetic Engineering and Biotechnology, Pathumthani, Thailand; ^5^ Intregated Biorefinery Excellent Center (IBC), School of Energy and Environment, University of Phayao, Muang Phayao, Thailand; ^6^ Department of Veterinary Technology, Faculty of Veterinary Technology, Kasetsart University, Bangkok, Thailand; ^7^ Department of Environmental Technology and Management, Faculty of Environment, Kasetsart University, Bangkok, Thailand

**Keywords:** corn stover, homogeneous catalyst, lignin fractionation, solvothermal based, optimization, phenolic compound

## Abstract

Fractionation of lignocellulosic is a fundamental step in the production of value-added biobased products. This work proposes an initiative to efficiently extract lignin from the corn stover using a single-step solvothermal fractionation in the presence of an acid promoter (H_2_SO_4_). The organic solvent mixture used consists of ethyl acetate, ethanol, and water at a ratio of 30: 25:45 (v/v), respectively. H_2_SO_4_ was utilized as a promoter to improve the performance and selectivity of lignin removal from the solid phase and to increase the amount of recovered lignin in the organic phase. The optimal conditions for this extraction, based on response surface methodology (RSM), are a temperature of 180°C maintained for 49.1 min at an H_2_SO_4_ concentration of 0.08 M. The optimal conditions show an efficient reaction with 98.0% cellulose yield and 75.0% lignin removal corresponding to 72.9% lignin recovery. In addition, the extracted lignin fractions, chemical composition, and structural features were investigated using Fourier transform infrared spectroscopy, thermogravimetric analysis, elemental analysis, and two-dimensional heteronuclear single quantum coherence nuclear magnetic resonance spectroscopy (2D-HSQC NMR). The results indicate that the recovered lignin primarily contains a β-O-4 linking motif based on 2D-HSQC spectra. In addition, new C–C inter-unit linkages (*i.e.,* β-β, and β-5) are not formed in the recovered lignin during H_2_SO_4_-catalyzed solvothermal pretreatment. This work facilitates effective valorization of lignin into value-added chemicals and fuels.

## Introduction

The development of alternative energy sources is important due to the depletion of fossil fuels and increased air pollution created by conventional vehicle combustion (Perera, 2017). Lignocellulosic materials from agricultural residual waste (such as rice straw, corn cob, and corn stover) have immense potential as replacements for fossil fuels and in reducing environmental problems. Several processes for utilizing these sustainable materials have been investigated globally (Ahorsu et al., 2018). Lignocellulosic material comprises mainly 35–50**%** cellulose, 20–35**%** hemicellulose, and 10–25**%** lignin (Kupczyk et al., 2019). Lignintraditionally considered a value-added product obtained during the fractionation process (Schutyser et al., 2018)—is classified as a three-dimensional amorphous polymer, consisting of p-coumaryl alcohol (4-hydroxycinnamyl), coniferyl alcohol (3-methoxy 4-hydroxycinnamyl), and sinapyl alcohols or 3,5-dimethoxy-4-hydroxycinnamyl (Mathew et al., 2018). The main lignin structure is characterized by various types of crosslinks. These structures are assembled *via* ether (such as β-O-4, α-O-4, and 4-O-5) and C-C bonds (Buranov and Mazza, 2008). The main functional groups include aliphatic, hydroxyl, carboxyl, carbonyl, phenolic, and methoxyl groups; the contents of these groups are higher in softwood lignin than in hardwood lignin (More, 2019). Typically, lignin can be combusted as a low-grade fuel to produce heat on an industrial scale. Recently, several studies have attempted to fractionate lignin from the biomass and to obtain high purity lignin-based products.

Fractionation of agricultural residues is a prerequisite for an integrated biorefinery. Thus far, studies have reported on the clean fractionation (CF) process utilizing a ternary organo-solvent mixture comprised of an organic solvent, water, and alcohol, in order to establish one-step isolation of the agricultural components with a relative high purity of each fraction. In CF, cellulose is enhanced in the solid pulp, and the hemicellulose-derived products are included in the aqueous phase, while a large amount of pure lignin is found in the organic fraction (Suriyachai et al., 2017). Lignin extracted *via* the organosolv process is called organosolv lignin. The extraction is called lignin’s potential source for depolymerization into beneficial biobased chemical products such as guaiacol, 4-methyl-guaiacol, 4-ethyl-guaiacol, 4-vinyl-guaiacol, vanillin, syringol, phenol, 4-methylphenol, 2,5-dimethylphenol, 4-hydroxy-2-methoxycinnamaldehyde, catechol, 3-methoxycatechol, toluene, p-xylene, and styrene (Zhang and Wang, 2020). Zhang et al. (2019) studied the fractionation of lignin from corncobs using a solvothermal process to separate the lignin for subsequent depolymerization. The optimal conditions were a temperature of 200°C maintained for 1 h using H_2_O/n-butanol (4:6, v/v). The results indicated that the maximum yields of extracted lignin and phenolic monomers were 87.1 and 19.5**%**, respectively. Ouyang et al. (2018) reported a two-step catalytic process for coupling the fractionation of woody biomass and the depolymerization of isolated lignin. Among various processes for the acid-catalyzed fractionation of biomass in methanol, the presence of sulfuric acid produced the best performance, with 70% of delignification. It was found that the degree of delignification impacted the yield in the depolymerization step due to the different repolymerization of lignin fragments. Inkrod et al. (2018) studied the CF of various lignocellulosic materials using a solvent mixture comprising methyl isobutyl ketone (MIBK), ethanol, and water using H_2_SO_4_. It was found that the efficiency of lignin isolation depended on the type of biomass. The separated lignin materials had different levels of purity and physiochemical properties. Meng et al. (2020) investigated the effect of various organosolv processes on the structure of woody biomass. At their optimal conditions, these organosolv processes facilitated the delignification of approximately 84, 85, and 52% ethanol (EtOH), co-solvent enhanced lignocellulosic fractionation (CELF), and γ-Valerolactone (GVL), respectively. Further, various organosolv processes produced different quantities of β-O-4 linkages. The preservation of these linkages is suitable for depolymerization to mono-aromatic compounds. Imman et al. (2015) studied the single-step solvothermal fractionation of rice straw (RS) with various organic solvents consisting of methyl isobutyl keone, ethyl acetate, toluene and diethyl ether, and acid promoter (H_2_SO4, HCl, and H_3_PO4) at 180 °C for 60 min. The results showed that the optimal conditions were found to be 0.05 M H_2_SO_4_ at 160°C for 1 h with a water/ethanol/ethyl acetate (62.5:25:12.5%) solvent mixture, resulting in the highest glucan yield of 71.4 wt%, hemicellulose (53.2 wt%), and lignin recovery (63.1 wt%) in the organic solvent phase.

Previous studies have shown that the CF process can modify depolymerization to obtain lignin monomers with high selectivity; however, characterization of the extracted lignin from corncob residue is unclear and poorly studied. According to our knowledge, the utilization of single-step solvothermal fractionation using ethyl acetate as an organic solvent under an acid promoter (H_2_SO_4_) has not been investigated with regard to extracting fractionated lignin from the corn stover. The use of ethyl acetate completed the drying process for isolated lignin. However, the side hydrolysis of ethyl acetate is required for further process optimization. This work aimed to enhance the yield and purity of separated lignin by solvothermal fractionation. The lignin fractionation process was carried out using ternary mixture solvents. A homogeneous catalyst was used as an acid promoter to enhance the recovery of lignin. Further characterization approaches (Klason lignin determination, elemental analysis, FT-IR, HPLC, TGA, GPC, 2DNMR-HSQC, XRD, BET, SEM, and pyrolysis GC-MS) were applied for a qualitative and quantitative analysis of the separated lignin, and to compare its properties with those of commercial lignin. The sulfuric acid catalyzed solvothermal process developed in this study enables efficient extraction of high-value organosolv lignin from the corn stover and the production of recovered lignin in the organic phase with low contamination by the other contents. The lignin characteristic data contributes to the development of lignin valorization in value-added applications.

## Materials and Methodology

### Materials

The corn stover was obtained from Ban so, Phayao, Thailand and dried at 70°C for 24 h in a hot air oven. Afterward, the dried corn stover was ground and sieved to a particles size between 0.5 and 2.0 mm. The final moisture content of the milled corn stover was 7**%**, measured as per the weight loss after drying in an oven (105°C) to achieve a constant weight within 5 h. For the experiments, the prepared corn stover was kept in sealed plastic bags at room temperature. The chemical composition was determined according to the analytical procedures of the National Renewable Energy Laboratory (Sluiter et al., 2008); dried corn stover was composed of cellulose (30%), hemicellulose (18%), lignin (35**%**), ash (10**%**), and extractives (7%). The commercial kraft lignin used in this work was provided by Sigma-Aldrich (low sulfonate content ∼4% and pH ∼10.5) for comparative study.

### Procedure of Lignin Isolation From Corn Stover Using Solvothermal Fractionation

Organosolv fractionation of raw corn stover was implemented in a stainless-steel reactor with a capacity of 600 ml. The corn stover was heated using an electric jacket with an inside thermocouple to measure the temperature (Parr Reactor 4,560, Parr Instrument Co., Moline, IL, United States). Initially, the screened corn stover sample (10 g) was mixed with the single-phase solvent mixture (100 ml) consisting of ethyl acetate: ethanol: water (30:25:45 v/v%) with various concentrations of H_2_SO_4_ acid promoter (0.05–0.1 M). The temperature and reaction time of each reaction ranged from 140 to 180°C and 30–60 min, respectively. The reaction time started once the target temperature had been reached. The heating profiles at different temperatures were provided to enable an understanding of their characteristics under fractionation conditions (Supplementary Figure S1). The reaction nitrogen gas (N_2_) was added in the reactor at a beginning pressure of 20 bar. The reaction was stirred at 100 rpm to maintain a homogeneous system. At the end of the reaction time, a water bath was used to quench the reaction immediately. The solid cellulose-enriched fraction was isolated using filtration and then washed with a mixture of ethyl acetate and 300 ml of water (1:2, v/v). Next, the liquid fraction was aggregated and settled in a separatory funnel. An appropriate amount of DI water was added to the liquid fraction. The slurry was then incubated at room temperature for 20 min to reach phase separation. Then, the aqueous phase (water, ethanol, acid promoter, and soluble products) was recovered. The separated organic phase (ethyl acetate) was dried at 105°C to obtain the separated lignin. Outputs were measured based on weight. The chemical composition was determined by applying the NREL method. The cellulose yield, cellulose purity, hemicellulose removal, lignin removal, and recovered lignin were calculated using the following equations:

$$\mathrm{Cellulose yield}\left(\text{\%}\right)=\frac{\left(\text{Cellulose remaining in solid pulp}\left(\text{mg}\right)\right)}{\left(\text{Cellulose content in raw corn stover }\left(\text{mg}\right)\right)}\;\times 100$$ (1)

$$\mathrm{Cellulose purity}\left(\text{\%}\right)=\frac{\left(\text{Cellulose remaining in solid pulp }\left(\text{mg}\right)\right)}{\left(\text{Total content in solid pulp}\left(\text{mg}\right)\right)}\text{ ×}100$$ (2)

$$\mathrm{Hemicellulose removal}\left(\text{\%}\right)=\frac{(\text{Hemicellulose content in raw corn stover }\left(\text{mg}\right))-\left(\text{Hemicellulose remaining in solid pulp}\left(\text{mg}\right)\right)}{\left(\text{Hemicellulose content in raw corn stover}\left(\text{mg}\right)\;\right)}\text{ ×}100$$ (3)

$$\mathrm{Lignin removal}\left(\text{\%}\right)\;=\frac{\left(\text{Lignin content in raw corn stover }\left(\text{mg}\right)\right)-\left(\text{Lignin remaining in solid pulp}\left(\text{mg}\right)\right)}{\left(\text{Lignin content in raw corn stover }\left(\text{mg}\right)\right)}\text{ × }100$$ (4)

$$\mathrm{Recovered lignin}\left(\text{\%}\right)=\frac{\left(\text{Weight of recovered lignin from organic phase}\left(\text{mg}\right)\right)}{\left(\text{Lignin content in raw corn stover}\left(\text{mg}\right)\right)}\text{ ×}100$$ (5)

### Statistical Design and Optimization for Recovered Lignin Using Response Surface Methodology

The experiment design and statistical analysis were implemented following the Box–response surface design method utilizing the Design Expert software (version 10.0.1). The experimental design was explored with three independent factors, based on a Box-Behnken design (BBD) with three levels. The three factors were reaction temperature (X_1_, 140–80°C), reaction time (X_2_, 20–60 min), and acid promoter concentration (X_3_, 0.05–0.1 M), with three coded levels for each factor (−1, 0, 1). Fifteen experiments with three replications at the center point were processed randomly. The assessed variance for individual factors was partitioned into offset, linear, interaction, and quadratic components. All parameters were evaluated utilizing a second-order polynomial equation,

$$\begin{array}{l} Y=\;\beta_{\text{0}}+\beta_{1}X_{1}+\beta_{2}X_{2}+\beta_{3}X_{3}+\beta_{12}X_{1}X_{2}\\ +\beta_{\mathrm{13}}X_{1}X_{3}+\beta_{23}X_{2}X_{3}+\;\beta_{11}X_{1}^{2}+\beta_{22}X_{2}^{2}+\beta_{33}X_{3}^{2} \end{array}$$ (6)

where *Y* is the predicted response; *X*_*1*_, *X*_*2*_, and *X*_*3*_ are the independent variables; *β*_*0*_ is a constant; *β*_*1*_ *, β*_*2*_, and *β*_*3*_ are the linear coefficients; *β*_*12*_, *β*_*13*_, and *β*_*2*3_ are the interaction coefficients; and *β*_*11*_, *β*_*22*_, and *β*_*33*_ are the quadratic coefficients. The fitted quadratic polynomial was utilized to obtain 3D surface plots of the correlations between the independent variables and response.

### Characterization of Isolated Solid Fraction

A scanning electron microscopy (SEM; JSM-6301F, JEOL, Japan) was used to examine the microstructure of raw corn stover and the remaining solid under optimal conditions. The electron beam energy was 20 Kv. The total surface area of the raw material and solid residues from solvothermal fractionation was determined using the Brunauer-Emmett and Teller (BET) method. Surface area and pore volume of the untreated and treated corn stover were determined using nitrogen adsorption/desorption isotherms in a surface area analyzer (TriStar II 3020, Micromeritics Co., United States). The degassing temperature and time were 30°C and 15 h, respectively. The crystallinity degrees of the native and isolated corn stover samples were determined using an X-ray diffraction (XRD) instrument (JDX-3530, JEOL, Japan), in which a Cu Kα radiation source was provided at 40 kV and 30 mA. The samples were implemented with a speed of 1°/min, in a range of 2θ = 5^o^ − 40°, and a step size of 0.004° at room temperature. The crystallinity index (CrI) was determined using the following equation:

$$\mathrm{CrI}=\;\frac{I_{002}-I_{\mathrm{amorphous}}}{I_{002}}\times 100$$ (7)

where I_002_ is the intensity of the crystalline portion of the biomass (cellulose) at 2 = 22.4 and I_amorphous_ is the peak of the amorphous portion, (such as cellulose, hemicellulose and lignin), at 2 = 18.0.

### Characterization of Lignin

#### Elemental Analysis

The elemental composition of the recovered lignin was quantified using an elemental analyzer CHNS-628 (LECO, Saint Joseph, MI, United States). Lignin samples were placed in a vacuum evaporator and dried at 60°C, 20 bars, to eliminate moisture. Afterward, a lignin sample (0.1 g) was placed in a container and its carbon, hydrogen, and nitrogen contents were measured. Then, for sulfur analysis (to determine the amount of sulfur), a lignin sample (0.2 g) was put in a ceramic boat furnace and incinerated at 1,350°C utilizing a sulfur IR cell.

### Fourier Transform Infrared Spectroscopy Analysis

The chemical structure of the recovered lignin was determined using Fourier transform infrared spectroscopy (FTIR) analysis with a PerkinElmer instrument (Waltham, MA, United States), based on the KBr pellet method to prepare the samples, and recording the region 4,000–400 cm^−1^ at 4 cm^−1^ resolution and 32 scans. The peaks of lignin were compared to the peaks of the standard functional groups.

### Thermogravimetric Analysis

The thermal stability of native and isolated lignin was detected using an instrument (Model TA Q50) from TA Instruments Inc. The specimens were heated at a specific rate (5°C/min) under a flow of nitrogen gas (20–35 ml/min). The specimen’s weight was controlled as a function of temperature. The recovered lignin (30–50 mg) was weighed in an aluminum pan and heated from ambient temperature to around 1,000°C to determine the total mass loss. The curve of weight loss versus temperature was generated from the recorded values and the derivative was calculated to determine the temperature corresponding to the maximum rate of weight loss.

### Gel Permeation Chromatography

The recovered lignin was analyzed using gel permeation chromatography (GPC) to determine the average molecular weight (M_w_) and polydispersity index (M_w_/M_n_). The samples were analyzed on a Jasco instrument equipped with an interface (LC-NetII/ADC) and a UV detector (254 nm). Lignin was dissolved in tetrahydrofuran (THF) at a concentration of 5% w/v. The conditions performed using the PolarGel-M 803 column and PolarGel-M guard were a flow rate of 0.5 ml/min at 40°C. Polystyrene standards (Sigma-Aldrich) in the range 266–55,000 g/mol were utilized for the calibration.

### Nuclear Magnetic Resonance Spectroscopy

The molecular structure of the recovered lignin was characterized using 2D-HSQC NMR (two-dimensional heteronuclear single quantum coherence-nuclear magnetic resonance spectroscopy). Lignin (0.05 g) was dissolved in DMSO-*d*_*6*_ (0.6 ml) and compared with commercial kraft lignin and recovered lignin samples using 2D-HSQC NMR analysis (NMR Bruker-AV500 MHz, Bruker, Germany). The experiment began with the determination of the ^1^H- and ^13^C-dimensions at 10–0 ppm and 220–5 ppm, respectively. Details setup of 2D-HSQC NMR conditions was published in our previous paper (Wanmolee et al., 2018; Wanmolee et al., 2021). Briefly, the correlation of ^1^H and ^13^C 2D spectra were recorded at 25°C using a 500 MHz spectrometer. A total number of scans of 1,024 points with a 1.5 s recycle delay and a total of 64 scans at 256 time increments were recorded for ^1^H and ^13^C dimensions, respectively. The 2D-HSQC NMR data set was processed with MestreNova software using a 90° shifted square sine-bell apodization window.

### Pyrolysis-Gas Chromatography–Mass Spectrometry

Pyrolysis-gas chromatography-mass spectrometry (Py-GC-MS) was used to determine the chemical composition of the recovered lignin. Pyrolysis of the recovered lignin was conducted at 500°C in an EGA/PY-3030D microfurnace pyrolyzer (Frontier Laboratories Ltd., Fukushima, Japan) connected to a GC 7820A system (Agilent Technologies, Inc., Santa Clara, CA), as well as an Agilent 5,975 mass-selective detector (Electron ionization of 70 eV). The dimensions of the DB-1701 column were an internal diameter of 30 m × 0.25 mm with a film thickness of 0.25 μm (J&W Scientific, Folsom, CA). The temperature of the oven was set up to increase at two distinct rates: 1) 20°C min^−1^ (from 50°C (1 min) to 100°C) and 2) 6°C min^−1^(starting from 50°C to 280°C within 5 min). Helium was used as the carrier gas (1 ml min^−1^). The released compounds were determined based on a comparison between their mass spectra and 1) those of the Wiley and NIST libraries, 2) the spectra reported in the literature (Zhan et al., 2017), and 3) whenever possible, with the retention times as well as mass spectra of authentic standards. The molar peak areas of the released lignin degradation’s individual products were estimated. The summed areas were normalized and the values of two replicates were averaged and presented as a percentage (Wanmolee et al., 2018).

## Results and Discussion

### Effect of Independent Variables on Cellulose Yield, Lignin Removal, and Lignin Recovery

All responses were evaluated based on the quality and quantity of the remaining solid fraction and the derived products in the liquid phase, which were dependent on the levels of independent factors in the solvothermal fractionation process. The optimized conditions were studied using RSM to determine the cellulose yield, hemicellulose removal, lignin removal, and recovered lignin.

Based on the design of the 15 experiments (Table 1), the results indicated that the aforementioned elements varied after the fractionation process in the ranges 74.5–97.3% (cellulose yield), 78.2–99.3% (hemicellulose removal), 52.5–76.1% (lignin removal), and 48.9–72.1% (recovered lignin). Increased reaction parameters (temperature, time, and acid concentration) led to increasing hemicellulose and lignin removal from the native corn stover into the liquid fraction, whereas the cellulose yield in the remaining solid decreased under severe conditions. The solid recovery was in range 40.9–70.2% based on weight. The cellulose purity in the solid fraction was in the range 52.9–60.1% (data not shown). The experiments were performed under optimal conditions to provide detailed information regarding the multiple variables identified based on their greatest effect on fractionation performance. The impact of reaction variables on the major components of the corn stover were determined using the Design Expert software.

**TABLE 1:** Effect of reaction factors on solid composition of solvothermal fractionation for lignin extraction using the response surface method (RSM).

		Factors		Responses (%)			
Run no.	Temp (°C)	Time (min)	Concentration (M)	Cellulose yield (%)^a^	Hemicellulose removal (%)	Lignin removal (%)^b^	Recovered lignin (%)^c^
1	140	20	0.075	97.3	78.25	52.50	49.40
2	180	20	0.075	84.0	98.96	59.60	56.10
3	140	60	0.075	74.5	89.93	70.0	67.12
4	180	60	0.075	80.4	99.12	75.80	71.70
5	140	40	0.050	76.2	76.02	66.10	63.00
6	180	40	0.050	77.6	97.22	74.30	72.10
7	140	40	0.100	86.3	89.94	68.20	64.80
8	180	40	0.100	75.3	99.10	76.12	70.80
9	160	20	0.050	90.0	82.27	53.30	48.90
10	160	60	0.050	80.0	89.16	71.10	68.40
11	160	20	0.100	94.0	91.57	57.40	53.60
12	160	60	0.100	81.4	99.37	70.30	67.10
13	160	40	0.075	84	92.04	72.20	70.00
14	160	40	0.075	84	92.04	72.20	70.00
15	160	40	0.075	84	92.04	72.20	70.00

a Based on relative content of cellulose in remaining pulp.

b Based on relative content of lignin in solid pulp compared with lignin content in raw material.

c Based on the weight of lignin in organic phase.

The optimization of the solvothermal fractionation conditions was determined for ranges in temperature (140–180°C), time (20 and 60 min), and acid concentration (0.05–0.1 M). All variable options were considered to maximize the responses of cellulose yield, lignin removal, and recovered lignin. The evaluation was analyzed based on the obtained results after the fractionation process (Table 1). The evaluated regression equations for the optimization of solvothermal fractionation considered the cellulose yield (Y_1_, %), lignin removal (Y_2,_ %), and recovered lignin (Y_3_, %) as functions of temperature (X_1_), time (X_2_), and acid concentration (X_3_). The predicted equation was fitted to a multiple second-order polynomial regression, defined by Eqs 1–3.

**Cellulose yield (%)** =

$$\begin{array}{l} {\text{Y}}_{1}=\left(-128\text{.}8\right)+\left(2{\text{.}854\text{X}}_{1}\right)-\left(2{\text{.}883\text{ X}}_{2}\right)+\left({1448\text{ X}}_{3}\right)-\left(\text{0}{\text{.}00930\text{ X}}_{1}^{2}\right)\\ +\left(\text{0}{\text{.}00943\text{ X}}_{3}^{2}\right)-\left({2272\text{ X}}_{3}^{2}\right)+\left(\text{0}{\text{.}012000\text{ X}}_{1}{\text{X}}_{2}\right)-\left(6{\text{.}180\text{ X}}_{1}{\text{X}}_{3}\right)-\left(1{\text{.}300\text{ X}}_{2}{\text{X}}_{3}\right) \end{array}$$ (8)

**Lignin removal (%)** =

$$\begin{array}{l} {\text{Y}}_{2}=\left(-19\text{.0}\right)+\left(\text{0}{\text{.}052\text{X}}_{1}\right)+\left(2{\text{.}0304\text{X}}_{2}\right)+\left({453\text{X}}_{3}\right)+\left(\text{0}{\text{.}000537\text{X}}_{1}^{2}\right)\\ -\left(\text{0}{\text{.}019850\text{X}}_{2}^{2}\right)-\left({01976\text{X}}_{3}^{2}\right)-\left(\text{0}{\text{.}000812\text{X}}_{1}{\text{X}}_{2}\right)-\left(\text{0}{\text{.}140\text{X}}_{1}{\text{X}}_{3}\right)-\left(2{\text{.}450\text{X}}_{2}{\text{X}}_{3}\right) \end{array}$$ (9)

**Recovered lignin (%)** =

$$\begin{array}{l} {\text{Y}}_{3}=\left(-86\text{.}1\right)+\left(\text{0}{\text{.}632\text{ X}}_{1}\right)+\left(2{\text{.}561\text{ X}}_{2}\right)+\left({856\text{ X}}_{3}\right)+\left(\text{0}{\text{.}000931\text{ X}}_{1}^{2}\right)\\ -\left(\text{0}{\text{.}021369\text{ X}}_{2}^{2}\right)-\left({3124\text{ X}}_{3}^{2}\right)-\left(\text{0}{\text{.}001325\text{ X}}_{1}{\text{X}}_{2}\right)-\left(1{\text{.}550\text{ X}}_{1}{\text{X}}_{3}\right)-\left(3{\text{.}000\text{ X}}_{2}{\text{X}}_{3}\right) \end{array}$$ (10)

As per the analysis of the variance (ANOVA) of the quadratic regression model, equations for the cellulose yield, lignin removal, and recovered lignin responses were statistically significant for the optimized fractionation of the corn stover at the confidence interval of 95%. Additionally, the R-squared values of all responses were greater than 0.9, which indicated a high level of accuracy of the regression model based on the comparison of the predicted and observed values. The prediction accuracy levels for the cellulose yield, lignin removal, and recovered lignin were 98.57, 99.21, and 99.13%, respectively. The effects of various parameters on the responses were also investigated. The details and discussions of the parameters for the three main responses (cellulose yield, lignin removal, and lignin yield) are provided in the next section.

**FIGURE 1:**
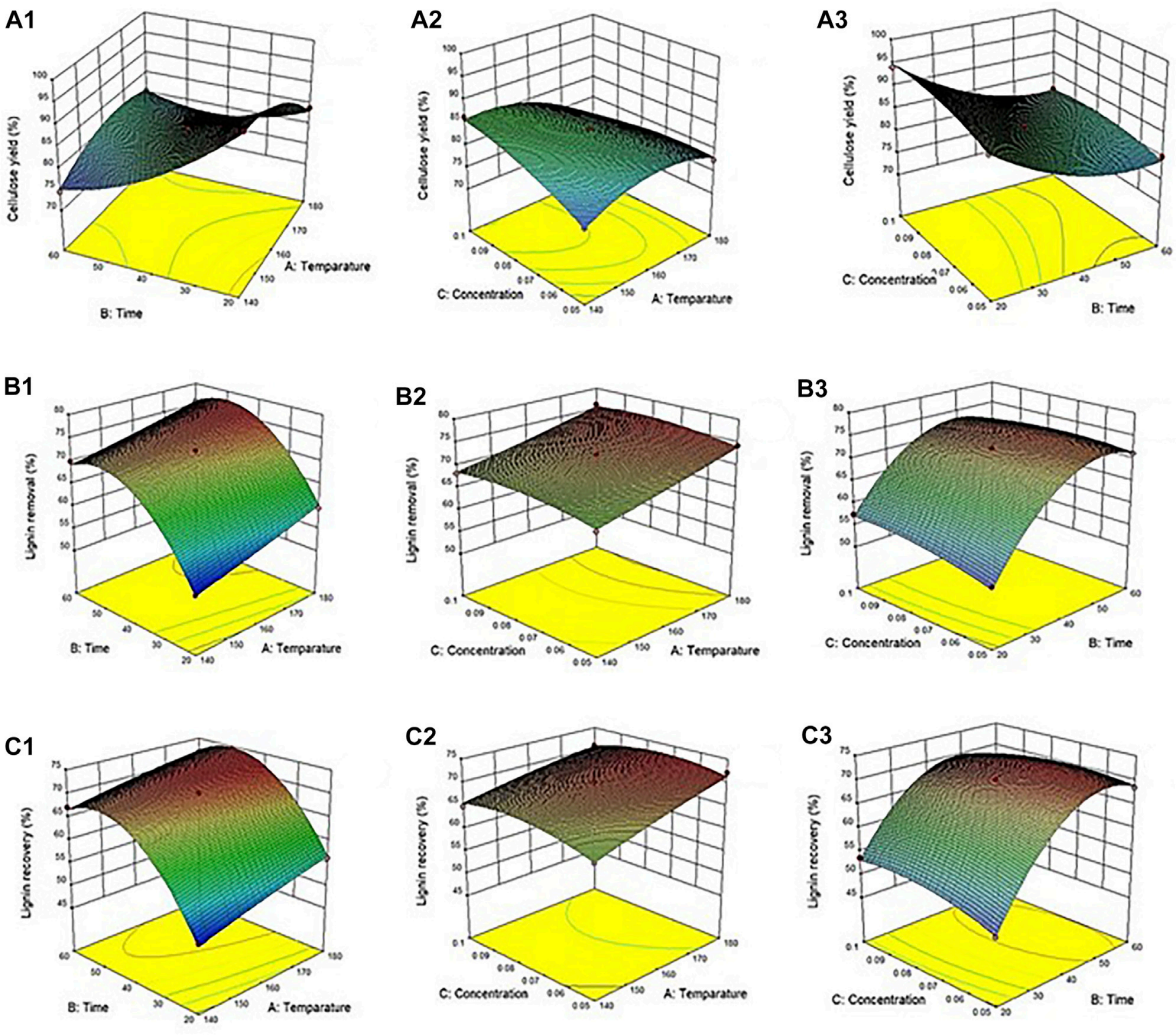
Response surface plot of the solvothermal fractionation process: Effect of various parameters on **(A)** cellulose yield **(B)** lignin removal and **(C)** recovered lignin.

Interactive effects of the variables (temperature, time, and acid concentration) on the cellulose yield from the corn stover were determined. The content of cellulose was directly dependent on the levels of various parameters (Figure 1A). The model was considered reliable based on the *p*-value at the 95% confidence level, with the *p*-value < 0.05. The linear terms (temperature, time, and acid concentration), quadratic terms (temperature and time), and interaction terms (temperature*time and temperature*concentration) all had significant effects on the cellulose yield (*p*-values < 0.05). Moreover, the relative coefficient of the sum of squares was summarized to determine the more important corresponding variable as shown in Supplementary Table S1. It was found that the reaction time had the most significant effect (highest coefficient value) on the cellulose yield.

In a previous report, the solvothermal fractionation cellulose yields of 50–60% were obtained at a high temperature of 180°C and a high ethanol concentration of 70% (Yedro et al., 2015). Harsh conditions, including high temperature, longer reaction time, and high acid concentration, result in the production of byproducts such as furfural, HMF, and other derivative compounds (Nopparat et al., 2018). The formation of the derivative compounds (furfural and HMF) critically decreased the high performance of the subsequent hydrolysis and fermentation steps (Kim, 2018). Further study of the product profiles in aqueous fraction is needed for valorization as co-products in the fractionation process.

The interactive effects of the variables (temperature, time, and acid concentration) on lignin removal from the corn stover were determined (Figure 1B). Lignin removal was dependent on the values of the parameters. The model was based on the *p*-value at a confidence interval of 95%. It was observed that the linear terms (temperature and time) and quadratic terms (time) both have a significant effect on lignin removal (*p*-value < 0.05). For example, the optimum condition (Run 8) had lignin removal of 76.1%, which was more efficient than that observed in a previous study (Hyväkkö et al., 2020). This phenomenon could have been due to a lower pretreatment temperature as well as a shorter exposure time. Lignin removal of 80.3% from sugarcane bagasse was examined during the pretreatment of ethanol organosolv at 160°C for 80 min (Li et al., 2018). Likewise, lignin (77.7%) was removed from the corn stover during the pretreatment of aqueous ammonia and hot water at 150°C for 60 min (Park et al., 2018). In this research, lignin decomposition was notably decreased under a combination of a relatively lower temperature and a short pretreatment time.

Efficient lignin recovery from lignocellulosic biomass using the organosolv process is a promising strategy in biorefining, since the organosolv lignin has high purity and can be modified to produce highly valuable products. In this study, the trend of recovered lignin initially depended on the percentage of lignin removal. This result indicates that harsh conditions can enhance the amount of recovered lignin (Figure 1C). Considering the influence of the parameters on recovered lignin, the model was based on the *p*-value at the confidence interval of 95%. The linear terms (temperature and time) and quadratic terms (time and concentration) both had substantial effects on lignin recovery (*p*-value < 0.05).

### Optimization of Reaction Parameters

According to the model equations for all target responses (Eqs. 4–6), the reaction parameters were optimized based on the final regression model to maximize the individual response. The highest cellulose yield of 100% was predicted at 140ºC for 20 min in the presence of 0.05 M acid. Harsher parameters were needed to maximize lignin removal and recovered lignin. The highest lignin removal (77.9%) was predicted at 180ºC for 49.5 min with 0.08 M acid promoter. Similar conditions at 180ºC for 49.5 min with 0.07 M acid were required for maximum recovered lignin (74.81%).

The optimal conditions were prioritized corresponding to the subsequent criteria: cellulose yield >80%, lignin removal >70%, and lignin recovery >70%. These conditions are of practical value in solvothermal fractionation to produce a large yield of cellulose while still achieving good lignin removal and lignin recovery in the organic phases. The regression model analysis indicated that the optimal predicted conditions to meet the particular requirements were 180ºC for 49.1 min with 0.08 M acid concentration in the solvent mixture of ethyl acetate: ethanol: water (30:25:45 v/v %). Accordingly, the experimental result obtained under these conditions generated a reaction efficiency of cellulose yield (98.0%), lignin removal (75.0%), and recovered lignin (72.9%). The experimental results were achieved using the criteria under the optimal conditions, thus verifying the accuracy of the model equations. Previous research studies have shown the maximum yields of recovered lignin in CF using various lignin solvents and different acid catalysts for fractionation of different lignocellulosic biomass, resulting in high reactivity and selectivity as shown in Supplementary Table S2 (Brudecki et al., 2013; Wen et al., 2013; Cheiwpanich et al., 2017; Nopparat et al., 2017).

### Characterization of Native Corn Stover and Remaining Solid After Solvothermal Fractionation Process

The physiochemical properties of the isolated solid after the solvothermal fractionation under the optimal conditions were compared with that of the native corn stover using several techniques (SEM, BET, and XRD).

**FIGURE 2:**
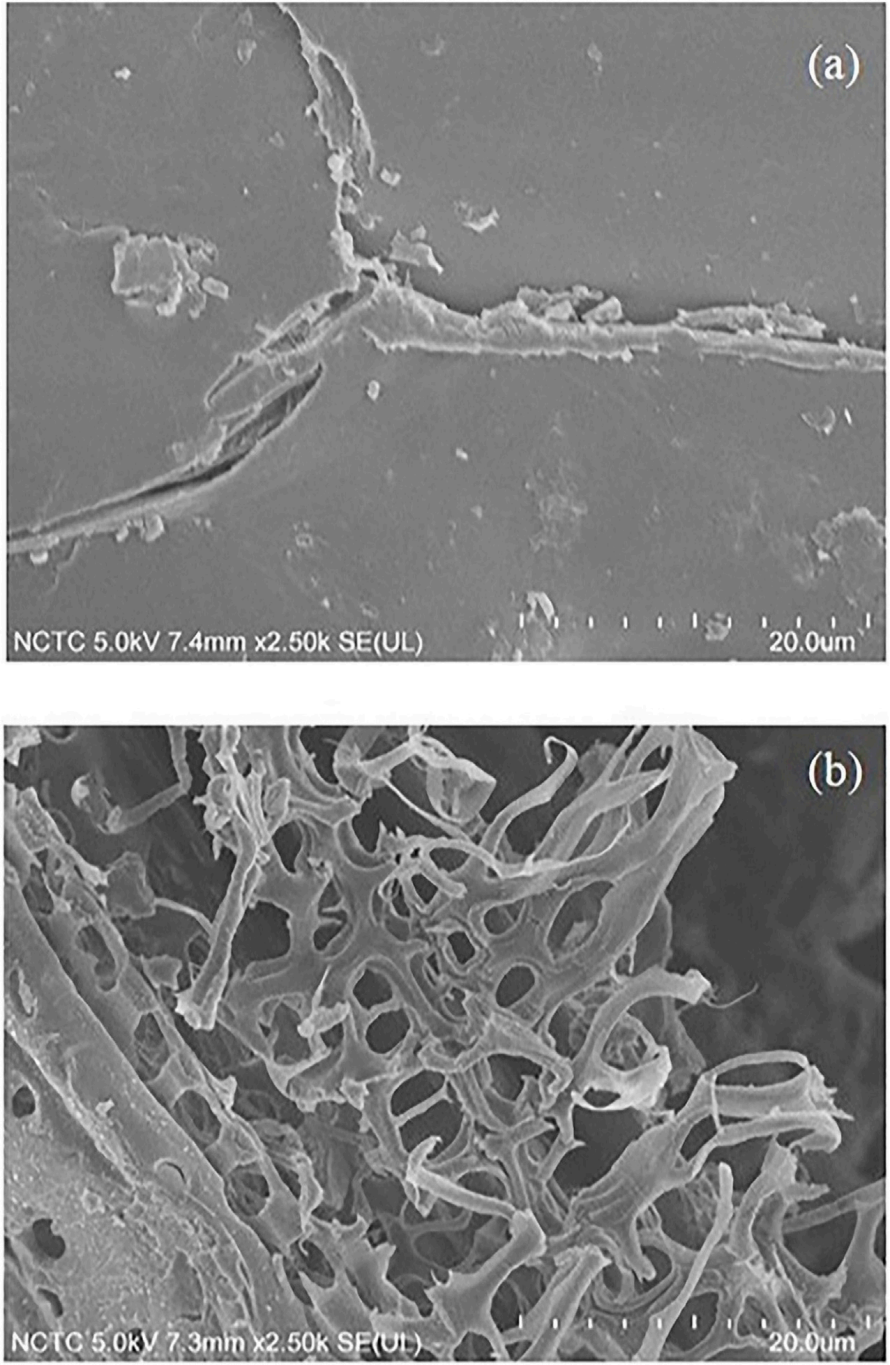
Scanning electron microscopy images of **(A)** native corn stover and **(B)** cellulose obtained after the solvothermal fractionation process under the optimal conditions at 0.075 M acid, 180°C, and 40 min.

The morphological appearance of the solid pulp is shown in Figure 2. The native corn stover was covered with wax and had a smooth, intact surface corresponding to the material’s highly ordered, intact structure. In addition, the isolated solid residue after solvothermal fractionation was considerably different from the native corn stover. The fractionation process resulted in the disruption of the surface and microstructure of the native corn stover. This disruption can be attributed to the elimination of waxy surface materials and the related hemicellulose, and lignin. As a result, very pure cellulose fibers were found in the isolated solid fraction, being obtained under the optimal conditions.

**FIGURE 3:**
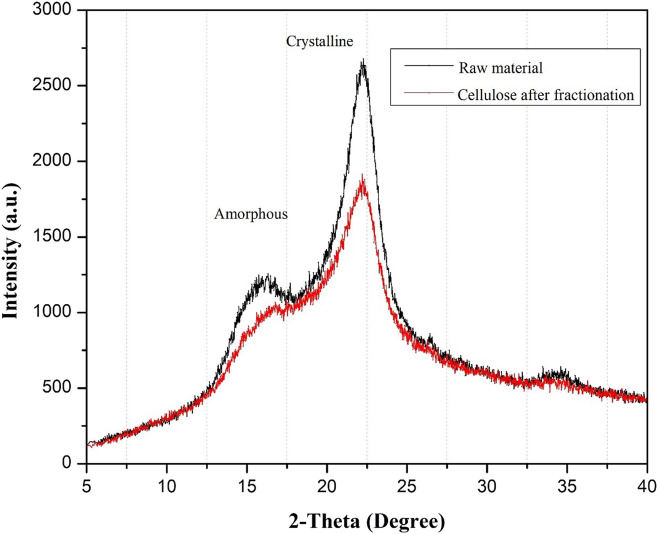
X-ray diffraction patterns of solid residue.

Additionally, the crystallinity of the solid fraction was examined using XRD (Figure 3). More decreased crystallinity (61.70%, based on the CrI) was detected in the isolated solid after solvothermal fractionation than in the native corn stover (76.13%). Crystallinity can decrease due to the elimination of amorphous hemicellulose and lignin from the biomass in the fractionation process. Furthermore, the harsh conditions can induce decrystallization of the cellulose fraction into an amorphous form, resulting in a substantial decrease in the CrI of the solid sample.

The structural modifications were further characterized using BET analysis (Supplementary Table S3). The solvothermal fractionation induced an expansion in the accessible surface area of the remaining pulp compared with the native corn stover. Substantial increases in the accessible surface area (2.21–7.32 m^2^/g) were observed after the fractionation reaction.

### Characterization of Lignin Fraction

The physiochemical properties of lignin recovered from solvothermal fractionation using the optimal conditions were compared to those of commercial kraft lignin using several techniques (FTIR, GPC, TGA, CHONS, proximate analysis, 2DHSQC-NMR, Py-GCMS, SEM, BET, and XRD). The composition of recovered lignin has been characterized previously to study the purity of Klason lignin. It was found that lignin recovered under the optimal conditions (180°C, 40 min, and 0.075 M acid) consisted of 89.6% Klason lignin and 0.76% acid-soluble lignin. Cross-contamination levels with sugar (less than 1.0%) and ash (0.16%) were minimal under these experimental conditions.

### Fourier Transform Infrared Spectroscopic Analysis of Commercial Kraft Lignin and Recovered Lignin

**FIGURE 4:**
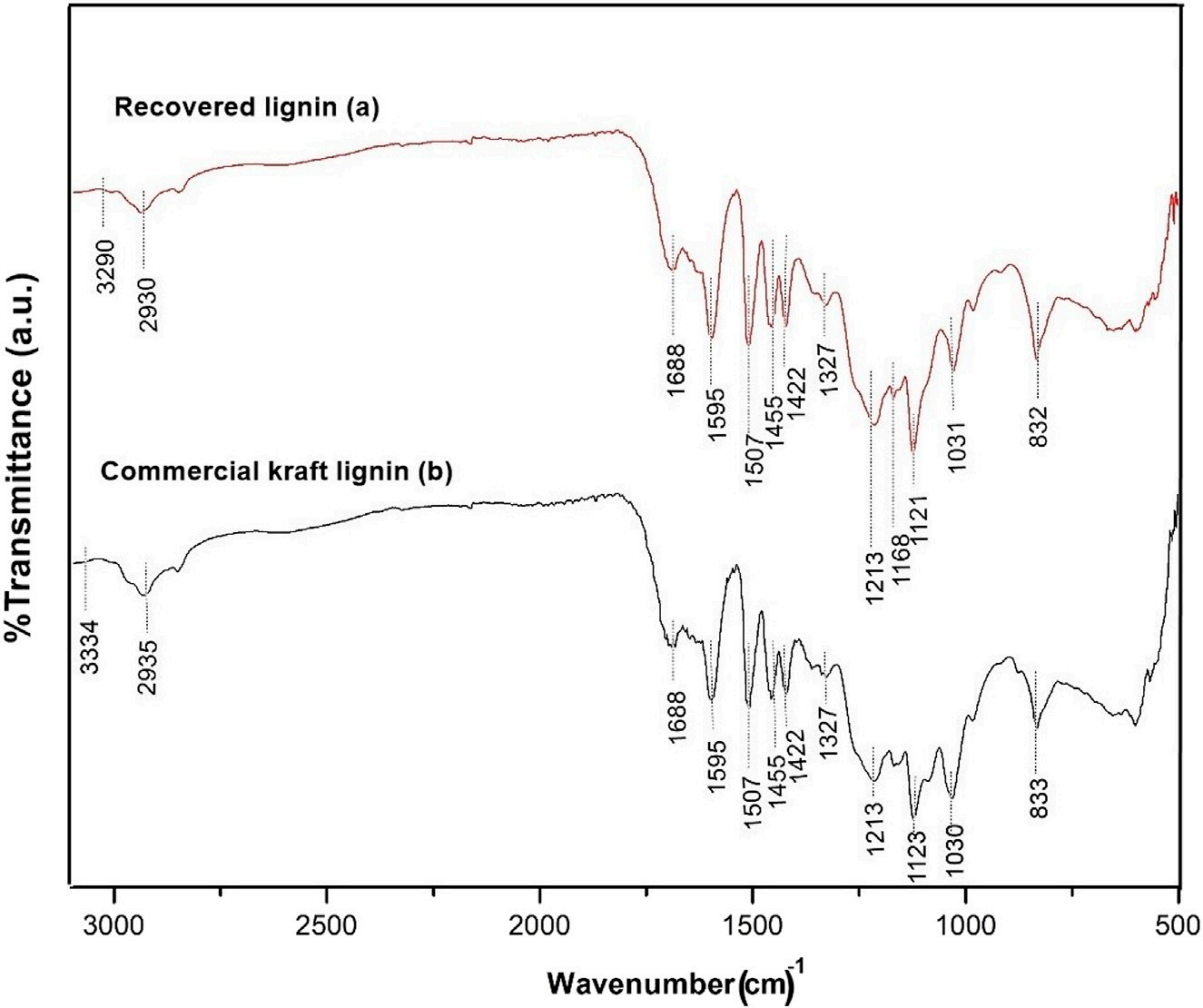
FTIR spectra of **(A)** recovered lignin and **(B)** commercial kraft lignin.

The chemical properties of the recovered lignin in the ethyl acetate phase using the optimal conditions were compared with those of commercial kraft lignin. The results of the FTIR analysis of the recovered lignin and commercial lignin are provided in Table 2; Figure 4. The extracted and commercial lignin were both characterized by the vibration of a hydroxyl group (O-H) of phenolic and aliphatic compounds at 3,290 and 3,334 cm^−1^ (Gonultas and Candan, 2018). The vibrations at 2,930 and 2,935 cm^−1^ were associated with (C-H_2_ and C-H_3_) vibrations in the aromatic methoxyl as well as in the methylene groups of side chains (Balan et al., 2019). The absorbance at 1,688 cm^−1^ corresponded to a carbonyl/carboxyl group. Variable peak intensity was observed in both samples at 1,422 cm^−1^ corresponding to the aromatic ring vibrations of a C-H group (Bykov, 2008). The intensity at 1,455 cm^−1^ was related to CH_3_ in an acetyl group, while the peak at 1,507 was assigned to the aromatic ring C=C stretching in lignin (Gonultas and Candan, 2018). The signal at 1,595 cm^−1^ corresponded to the quantity of the phenyl-propane skeleton present in the samples (Dominguez-Robles et al., 2017). The vibrations at 1,121–1,213 cm^−1^ corresponded to the C-O stretching of the syringyl (S) and guaiacyl (G) units (Quoc Lam et al., 2001). The peaks at 1,030 and 1,031 cm^−1^ were associated with the C–O vibrations (Oliveira et al., 2009). C–O deformation of the primary alcohols and C–H stretching (unconjugated) can also be observed (Klein et al., 2018). In addition, the absorbance bands at 832–833 cm^−1^ were associated with the outcome of plane C–H vibrations of the guaiacyl units (G) (Raspolli Galletti et al., 2015).

**TABLE 2:** The relative functional groups based on FTIR analysis of commercial kraft lignin and recovered lignin obtained from solvothermal fractionation process under the optimal conditions.

Order	Commercial kraft lignin	Recovered lignin	Bond		Wavenumber, (cm^−^1)	Functional group
1	3,334	3,290	O-H stretch	3,500–3,200		Alcohol, phenol
2	2,935	2,930	C-H stretch	3,000–2,930		C–H stretch in methyl and methylene groups
3	1,688	1,688	C=O	1760–1,665		Carbonyl
4	1,595	1,595	C-C stretch (in-ring)	1,605–1,593		Aromatic skeleton vibrations (S, G) and C-O stretching
5	1,507	1,507	C=C stretching	1,550–1,475		Aromatic
6	1,422	1,422	C–H in plane deformation (G, S)	1,430–1,422		Aromatics
7	1,455	1,455	C–H deformation (lignin)	1,470–1,370		Aromatics
8	1,213, 1,123, 1,327	1,327, 1,213, 1,168, 1,121	C-O stretch	1,320–1,000		Alcohol, carboxylic acid, ester, ether
9	1,030	1,031	C–O vibrations	1,030.1031		C–O deformation in primary alcohols, C–H stretching (unconjugated)
10	833	832	C–H	832–817		C–H out of plane (G-units)

### Molecular Weight Distribution of Commercial Kraft Lignin and Recovered Lignin

The molecular weight distribution of the lignin samples is demonstrated in Table 3. GPC analysis was used to evaluate the average molecular weight (M_w_), number-average molecular weight (M_n_), and polydispersity index (PDI) of the recovered lignin and these were compared with those of commercial kraft lignin. The average M_w_ and M_n_ values of the recovered lignin were 1,561 and 1,315 Da, respectively, with a PDI of 1.18. The results indicated that the particle size of the recovered lignin was not influenced by the fractionation process. In comparison, the average M_w_ and M_n_ values of commercial kraft lignin were 1,399 and 1,046 Da, respectively, with a PDI of 1.34. The commercial lignin had a higher distribution of molecular weight than the recovered lignin. A previous report demonstrated that lignin recovered from BioChoice Lignin (BCL) *via* the organosolv fractionation process using a ternary mixture (ethyl acetate, 1:1 ethyl acetate/petroleum ether, petroleum ether) had a PDI of 1.12, similar to the PDI of the lignin recovered from the organic phase, which was not significantly different from the PDI of the original lignin (3.33) (Jiang et al., 2016). The recovered lignin obtained under the optimal conditions using the solvothermal conversion process in a previous report had a PDI of 1.66, which was lower than that of commercial lignin (2.39) (Liu et al., 2019). Unlike high molecular weight lignin, low molecular weight lignin can be easily utilized in biochemical and other value-added products in the biorefinery industry (Zhang et al., 2020).

**TABLE 3:** Molecular weight analysis of the commercial kraft lignin and recovered lignin.

Order	Sample name	Mw^a^ (g/mol)	Mn^b^ (g/mol)	Mw/Mn (PDI)^c^
1	Commercial kraft lignin	1,399	1,046	1.34
2	Recovered lignin	1,561	1,315	1.18

a Weight-average molecular weight (Mw).

b Number-average molecular weight (Mn).

c Polydispersity index (PDI).

### Proximate Analysis of Commercial Kraft Lignin and Recovered Lignin

The composition of the recovered lignin under the optimal conditions was characterized based on proximate analysis and compared to the commercial kraft lignin (Table 4). The composition of the samples included volatile materials (VM), fixed carbon (FC), moisture, and ash (wt%, on a dry basis or d.b%). The recovered lignin and commercial kraft lignin had the same range of elemental contents. The proximate analysis of recovered lignin indicated high contents of volatile matter (61.2–67.6 wt%), fixed carbon (24.5–35.7 wt%), moisture (0.8–2.0 wt%), and ash (1.0–7.0 wt%). The ash content in the recovered lignin was slightly higher than that in the commercial kraft lignin apparently because of the high amounts of sulfur used as a promoter in the solvothermal fractionation process (Javad et al., 2014).

**TABLE 4:** Proximate and elemental analysis of the commercial kraft lignin and recovered lignin.

Proximate analysis^a^ (%)				Elemental analysis^a^ (%)					
Optimal condition	Volatile materials (d.b%)	Fixed carbon (d.b%)	Moisture (%)	Ash (d.b%)	C	H	N	O	S
Commercial kraft lignin	61.21	35.68	2.02	1.00	61.43	5.61	0.81	30.99	0.15
Recovered lignin	67.60	24.56	0.84	7.00	63.08	5.20	0.70	30.62	0.54

a Dry basis (db, wt%).

### Thermal Decomposition of Commercial Kraft Lignin and Recovered Lignin

**FIGURE 5:**
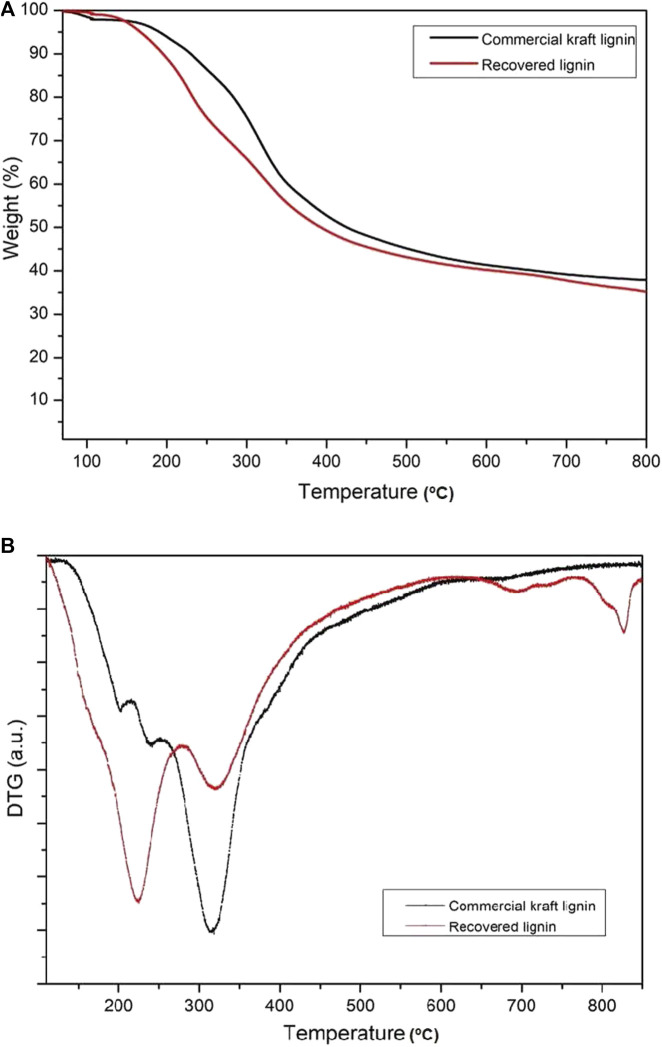
**(A)** TG (weight loss) and **(B)** DTG (derivatives of weight loss) curves of commercial kraft lignin and recovered lignin after solvothermal fractionation.

Thermogravimetric analysis (TGA) or thermal stability is important in the examination of the thermal properties of the recovered lignin and commercial kraft lignin samples as it can identify potential relationships between degradation and chemical structure. The thermal stability levels of the recovered and commercial lignin were assayed using thermogravimetry (TG) and differential thermogravimetry (DTG), as shown in Figure 5. Overall, the degradation of the recovered lignin was divided into four stages. The first stage was observed at ∼106.7°C, considered the dehydration process. In fact, the water content in the recovered lignin is eliminated at temperatures above 100°C, indicating a strong interaction between the water molecules and hydroxyl groups. The second stage included the decomposition of a number of volatile substances, such as CO, CO_2_, and CH_4_, due to the breaking of the side chains concomitant to a temperature increase from 150 to 350°C (Abdelkhalik et al., 2018). The highest lignin decomposition temperatures of recovered and commercial kraft lignin were estimated to be 224 and 318°C, respectively, due to the breakdown of interunit links and breaking of various bonds of weak internal linkages in the phenolic and organic products during the temperature considering the high content of b′-O-4′ linkages (Satari et al., 2019). According to the degradation in the second stage, the weight loss of commercial kraft lignin was approximately 41% and that of recovered lignin was approximately 46%. This result indicates the higher thermal stability of commercial kraft lignin. The third stage was decomposition above 400°C, mainly due to the degradation of aromatic rings and breaking of the C-C linkages, majorly the 5–5′ and b′-b′ linkages (Gao et al., 2018). Finally, the degradation of the aromatic rings in the demethoxylation reaction (methoxy groups) was observed at temperatures ranging from 450 to 550°C (Kawamoto, 2017). This degradation process involves fragmentation of the interunit linkages and release of the monomers and derivatives of the aromatic compounds into the vapor phase. In addition, the weight loss rate of lignin samples is demonstrated in the DTG graph (Figure 5B). The maximum weight loss rate in commercial kraft lignin occurred at 325°C, whereas that of recovered lignin occurred at two different temperatures (232 and 334°C). These results also demonstrated that commercial kraft lignin is more thermally stable than the recovered lignin as a representative of organosolv lignin. This could be because the organosolv fractionation provides a higher number of weak linkages (e.g., β-O-4) in isolated lignin as compared to kraft lignin (Cherif et al., 2020).

### NMR Spectra of Recovered Lignin

**FIGURE 6:**
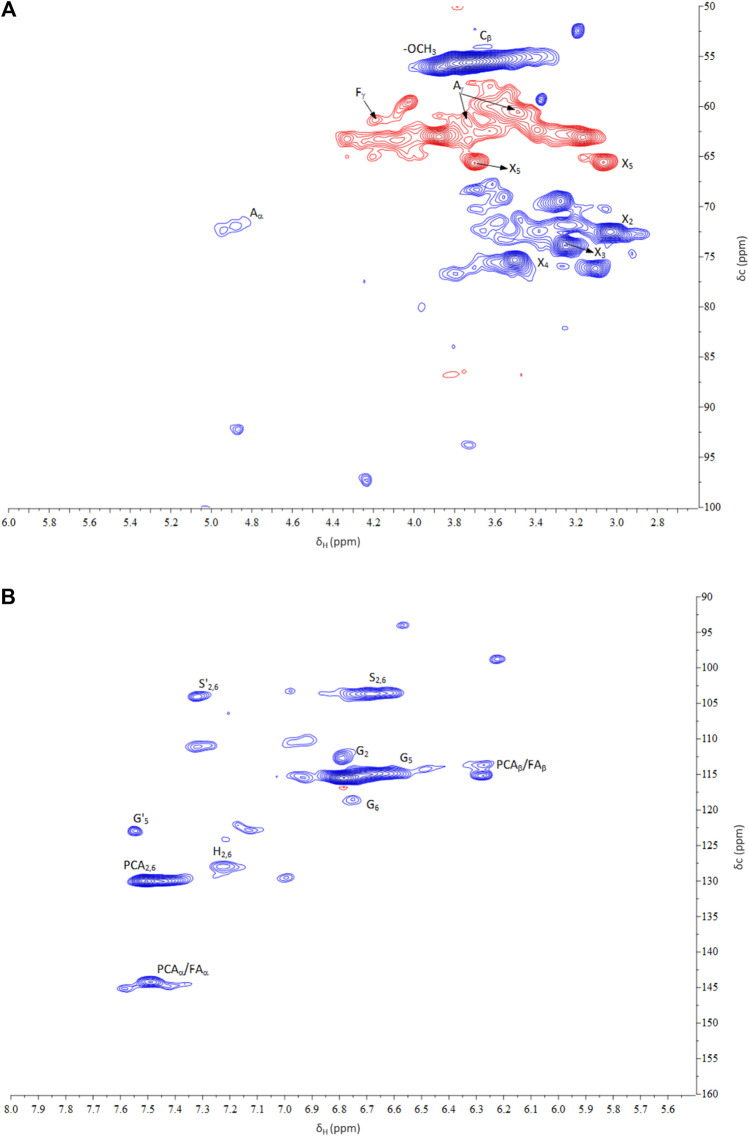
2DHSQC-NMR spectra of **(A)** side-chain and **(B)** aromatic regions of recovered lignin.

**FIGURE 7:**
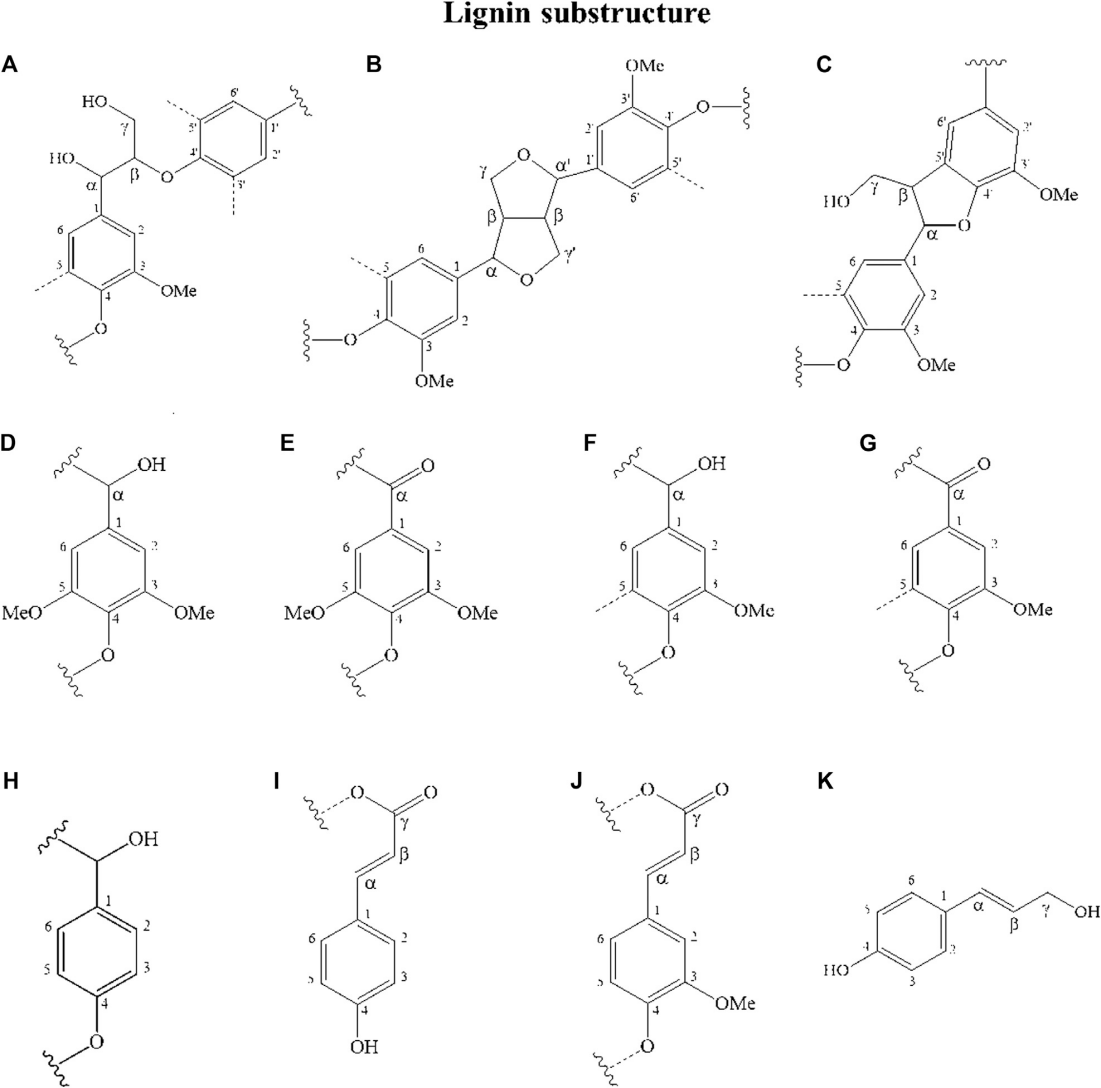
Lignin substructures detected in the HSQC spectra of recovered lignin samples: **(A)** β-O-4 linkages; **(B)** resinol structures formed by β-β; **(C)** phenylcoumaran substructures formed by β-5; **(D)** syringyl unit; **(E)** oxidized syringyl unit bearing a carbonyl group at Cα (phenolic); **(F)** guaiacyl unit; **(G)** oxidized guaiacyl unit bearing a carbonyl group at Cα (phenolic); **(H)** p-hydroxyphenyl unit; **(I)** p-coumarate; **(J)** ferrulate; and **(K)** p-hydroxycinnamyl alcohol.

Lignin obtained from the corn stover under the optimal conditions was analyzed using 2DHSQC-NMR. The assignments of correlation signals in the spectra are provided in Supplementary Table S4 based on the correlation signals described in the literature (Jiang et al., 2018). Figures 6, 7 show the 2DHSQC-NMR of recovered lignin and the lignin substructures, respectively. The side-chain region (δ_C_/δ_H_ 50.0–100.0/2.0–6.0) of recovered lignin is shown in Figure 6A. The data indicated the presence of interunit linkages and terminal structures in lignin and in LCC (lignin–carbohydrate complexes). The most common lignin linkage structures, such as those formed by the substructures of the β–aryl structures (β-O-4, A), resinol (β-β, B), and phenylcoumaran (β-5, C), were evident in the recovered lignin. The most well-known cross-signals represent methoxyl groups (-OMe) and side chains in the β-O-4 substructures. The C_β_–H_β_ correlation signal of the side chain in the β-O-4 linkage was attributed to guaiacyl linked with the *p*-hydroxyphenyl units (G/H) (Sun et al., 2011). Added to the interunit lignin linkages was that the strong signals of the LCC linkages from C_2_–H_2_ (X_2_), C_3_–H_3_ (X_3_), C_4_–H_4_ (X_4_), and C_5_–H_5_ (X_5_) were found at δ_C_/δ_H_ 72.69/3.05, 73.90/3.24, 75.54/3.49, 63.25/3.15, and 3.85, respectively (Wen et al., 2012; Fernández-Costas et al., 2014). Furthermore, the signal at δ_C_/δ_H_ 61.43/4.17 corresponding to C_γ_–H_γ_ in *p*-hydroxycinnamyl alcohol was detected in the recovered lignin. This monolignol is an intermediate in the biosynthesis and may represent the polymeric structures of *p*-coumaryl alcohol or *p*-coumarate (*p*CA).

The ^13^C–^1^H correlations δ_C_/δ_H_ 100.0–150.0/6.0–8.0 (Figure 6B) were associated with the aromatic/unsaturated side chain region of the S-, G-, and H- units, and the *p*CA and FA pendant units corresponded to lignin. The typical G monolignol structures were determined by various correlation signals of C_2_–H_2_ (G_2_), C_5_–H_5_ (G_5_), and C_6_–H_6_ (G_6_). The S and H unit cross-signals were associated with C_2,6_–H_2,6_. The presence of the C_α_-oxidized S and G units referring to C_2,6_–H_2,6_ (S′) and C_6_-H_6_ (G′) was detected. The *p*CA monomers corresponding to the C_2,6_–H_2,6_ and C_3,5_–H_3,5_ correlation peaks were identified; an overlap of the latter signal with the G_5_ signals from the guaiacyl units in corn stover lignin was observed. The unsaturated C_α_–H_α_ and C_β_–H_β_ correlations of *p*CA were also detected in this region. In addition to the signals of the *p*CA units, minor signals of ferrulate (FA) were detected. The signal of C_6_–H_6_ (FA_6_) confirmed the existence of these moieties, whereas the signals of unsaturated C_α_–H_α_ and C_β_–H_β_ correlations overlapped with the signals of the *p*CA units. These results showed a high consistency regarding the reported NMR data for the *p*CA units and were confirmed using Py-GC/MS (Kim et al., 2017).

### Identification of Composition of Commercial Kraft Lignin and Recovered Lignin

The Py-GC/MS analysis indicated more substantial variations in the lignin composition of lignin recovered under optimal conditions compared to that of commercial kraft lignin. The identities and relative abundance of the released lignin-derived phenolic compounds and relative abundance of the H, G, and S-lignin units and S/G ratios are illustrated in Supplementary Table S5. The results showed that the commercial kraft lignin contained G-units (90.4%) almost exclusively, with a minor number of H-units (9.6%), whereas the recovered lignin had a higher amount of G- (53.5%) than S- (33.1%) units, leading to an S/G ratio of approximately 0.7.

Higher amounts of 4-vinylguaiacol (14.8%) and 4-vinylphenol (2.5%) were released from the recovered lignin sample compared with the commercial kraft lignin; these elements accounted for the high percentage of the G- and H-type units found in these samples. Nonetheless, notably, 4-vinylguaiacol, and 4-vinylphenol may also have occurred from FA and *p*-coumarates (*p*CA), respectively upon decarboxylation during pyrolysis as usually arises during the pyrolysis of grass lignins (Del Río et al., 2012; Del Río et al., 2015; Del Río et al., 2017). Remarkably, these lignin samples released high amounts of C_α_-oxidized phenolic compounds derived from the H-, G-, and S-lignin units, including aromatic aldehydes (3,4-dimethoxybenzaldehyde, vanillin, and syringaldehyde) and ketones (acetoisovanillone, acetosyringone, guaiacylacetone, and andpropiosyringone). Considerable amounts of C_α_-oxidized compounds released from the recovered lignin clearly indicated the high extent of oxidation compared with the commercial kraft lignin.

Interestingly, a series of fatty acids (FatAc), such as hexadecenoic acid, 6-octadecenoic acid, 1-heptadecanecarboxylic acid, octadecanoic acid, ethyl ester, docosanoic acid, ethyl ester, and n-hexadecanoic acid, accounted for 10.9% of all released compounds that emerged during pyrolysis, indicating the high aliphatic character of the recovered lignin sample. Nevertheless, no additional structural information was obtained using Py-GC/MS from the sample due to the technique’s current limitations, such as decarboxylation of the carboxylic moieties (Jose et al., 1998; Ana et al*.*, 1998).

Linkages between the lignin units, (such as β-O-4/α-O-4) were cleaved corresponding to a range in temperature (150–350°C) because of the relatively low bond dissociation energy to produce the alkoxyls of the G-type substructures and the hydroxyl groups of the H-type substructures as the major products.

## Conclusion

A modified one-step organosolv fractionation method of lignin fractionation from corn stover was investigated. Under the optimum conditions, the method enhanced lignin removal (75.0%) from the solid phase and recovered lignin in the organic phase (72.9%). The fractionation process provides highly pure recovered lignin (89.6%) under the optimal conditions. An FTIR analysis showed variations in the bonding pattern and functional groups. These distinguishable chemical functionalities are substantial components for lignin product applications. The thermal degradation of the recovered lignin was due to the breakdown of the interunit links and breakage of various bonds of the lignin structure’s weak internal linkages. GPC analysis recorded a low molecular weight for the recovered lignin containing high levels of phenolic hydroxyls, methoxyl function groups, and β-O-4/α-O-4 linkages. The Py/GCMS analysis illustrated the predominance of G- (53.5%) units over S- (33.1%) units. A series of fatty acids was obtained during pyrolysis. This work demonstrates a novel solvothermal-based lignin fractionation of lignocellulosic biopolymers with improved reaction efficiency and selectivity for potential application in an integrated biorefinery.

## Data Availability

The original contributions presented in the study are included in the article/Supplementary Material, further inquiries can be directed to the corresponding author.
