# Supplementary material for: Solvothermal-Based Lignin Fractionation From Corn Stover: Process Optimization and Product Characteristics
Source: Front Chem. 2021 Aug 5;9:697237. doi: 10.3389/fchem.2021.697237 (PMC8374146; doi:10.3389/fchem.2021.697237)
Supplement: Supplementary file 1 [file DataSheet1.docx]

**Solvothermal-Based Lignin Fractionation from Corn Stover: Process Optimization and Product Characteristics**

**Punjarat Khongchamnan^1^, Wanwitoo Wanmolee^2^, Navadol Laosiripojana^3,4^, Verawat Champreda^4^,** **Nopparat Suriyachai^4,5^, Torpong Kreetachat^1,5^, Chainarong Sakulthaew^6^, Chanat Chokejaroenrat^7^, Saksit Imman,^1,5*^**

^1^School of Energy and Environment, University of Phayao, Tambon Maeka, Amphur Muang Phayao 56000, Thailand

^2^National Nanotechnology Center, National Science and Technology Development Agency, 111 Thailand Science Park, Paholyothin Rd., Klong Laung, Pathumthani 12120, Thailand

^3^The Joint Graduate School for Energy and Environment (JGSEE), King Mongkut’s University of Technology Thonburi, Prachauthit Road, Bangmod, Bangkok 10140, Thailand

^4^BIOTEC–JGSEE Integrative Biorefinery Laboratory, National Center for Genetic Engineering and Biotechnology, Innovation Cluster 2 Building, Thailand Science Park, Khlong Luang, Pathumthani 12120, Thailand

^5^Intregated Biorefinery Excellent Center (IBC), School of Energy and Environment, University of Phayao, Tambon Maeka, Amphur Muang Phayao 56000, Thailand

^6^Department of Veterinary Technology, Faculty of Veterinary Technology, Kasetsart University, Bangkok, Thailand

^7^Department of Environmental Technology and Management, Faculty of Environment, Kasetsart University, Bangkok, Thailand

*** Correspondence:**Corresponding Author
saksit.im@up.ac.th (S. Imman)

**Supplementary information**


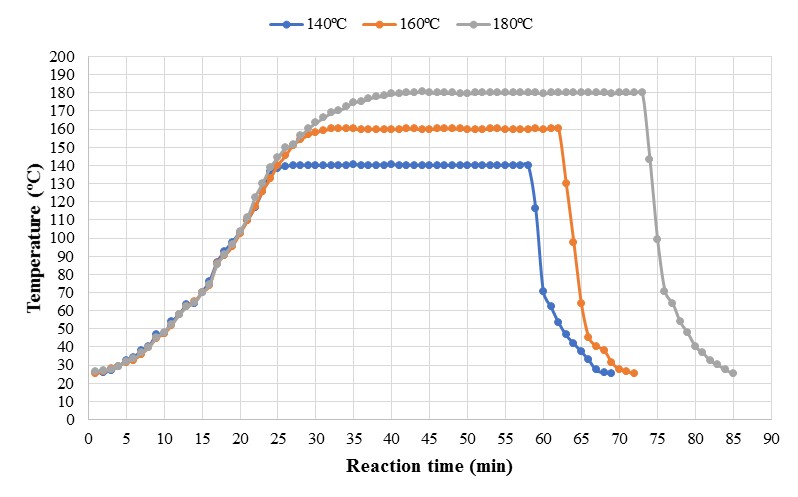


**Figure S1.** The heating profile of various temperature with varying residence time for 30 min.

**Table S1.** ANOVA analysis of various responses.

1. **Cellulose yield**
2. **Lignin removal**

| **Source** | | | **Sum of Squares** | **Degree of freedom** | | **Mean Square** | **F-value** | **p- value** |
| --- | --- | --- | --- | --- | --- | --- | --- | --- |
| **Linear term** | | |  |  | |  |  |  |
| **Acid conc.** | | | **6.516** | **1** | | **6.516** | **13.20** | **0.015** |
| **Temp.** | | | **105.270** | **1** | | **105.270** | **213.17** | **0.000** |
| **Time** | | | **518.420** | **1** | | **518.420** | **1049.82** | **0.000** |
| **Quadratic term** | | |  |  | |  |  |  |
| Acid conc.× Temp | | | 0.020 | 1 | | 0.020 | 0.04 | 0.850 |
| **Acid conc. ×Time** | | | **6.003** | **1** | | **6.003** | **12.16** | **0.018** |
| Temp ×Time | | | 0.423 | 1 | | 0.423 | 0.86 | 0.397 |
| **Interaction term** | | |  |  | |  |  |  |
| **Acid conc.× Acid conc** | | | **5.632** | **1** | | **5.632** | **11.40** | **0.020** |
| Temp × Temp | | | 0.171 | 1 | | 0.171 | 0.35 | 0.582 |
| **Time × Time** | | | **232.776** | **1** | | **232.776** | **471.38** | **0.000** |
| *Total* | | 876.172 | | 14 |  | |  |  |
| R-Square = 99.72% | R-Square  (predict)= 95.49% | | | R-Square (adjust) = 99.21% | S= 0.702 | |  |  |
|  |  | | |  |  | |  |  |

| **Source** | | **Sum of Squares** | | **Degree of freedom** | | **Mean Square** | **F-value** | **p- value** |
| --- | --- | --- | --- | --- | --- | --- | --- | --- |
| **Linear term** | |  | |  | |  |  |  |
| **Acid conc.** | | | **21.912** | **1** | | **21.912** | **34.98** | **0.002** |
| **Temp.** | | | **35.955** | **1** | | **35.955** | **57.39** | **0.001** |
| **Time** | | | **300.125** | **1** | | **300.125** | **479.07** | **0.000** |
| **Quadratic term** | | |  |  | |  |  |  |
| **Acid conc.× Temp** | | | **38.192** | **1** | | **38.192** | **60.96** | **0.001** |
| Acid conc. ×Time | | | 1.690 | 1 | | 1.690 | 2.70 | 0.161 |
| **Temp ×Time** | | | **92.160** | **1** | | **92.160** | **147.11** | **0.000** |
| **Interaction term** | | |  |  | |  |  |  |
| **Acid conc× Acid conc** | | | **7.445** | **1** | | **7.445** | **11.88** | **0.018** |
| **Temp × Temp** | | | **51.096** | **1** | | **51.096** | **81.56** | **0.000** |
| **Time × Time** | | | **52.478** | **1** | | **52.478** | **83.77** | **0.000** |
| *Total* | | 612.937 | | 14 |  | |  |  |
| R-Square = 99.49% | R-Square  (predict)= 91.82% | | | R-Square (adjust) = 98.57% | S= 0.791 | |  |  |
|  |  | | |  |  | |  |  |
|  |  | | |  |  | |  |  |

| **Source** | | | **Sum of Squares** | **Degree of freedom** | | **Mean Square** | **F-value** | **p- value** |
| --- | --- | --- | --- | --- | --- | --- | --- | --- |
| **Linear term** | | |  |  | |  |  |  |
| Acid conc. | | | 1.901 | 1 | | 1.901 | 3.29 | 0.129 |
| **Temp.** | | | **86.988** | **1** | | **86.988** | **150.60** | **0.000** |
| **Time** | | | **549.793** | **1** | | **549.793** | **951.82** | **0.000** |
| **Quadratic term** | | |  |  | |  |  |  |
| Acid conc.× Temp | | | 2.402 | 1 | | 2.402 | 4.16 | 0.097 |
| **Acid conc. ×Time** | | | **9.000** | **1** | | **9.000** | **15.58** | **0.011** |
| Temp ×Time | | | 1.124 | 1 | | 1.124 | 1.95 | 0.222 |
| **Interaction term** | | |  |  | |  |  |  |
| **Acid conc.× Acid conc** | | | **14.076** | **1** | | **14.076** | **24.37** | **0.004** |
| Temp × Temp | | | 0.512 | 1 | | 0.512 | 0.89 | 0.390 |
| **Time × Time** | | | **269.759** | **1** | | **269.759** | **467.02** | **0.000** |
| *Total* | | 930.616 | | 14 |  | |  |  |
| R-Square = 99.69% | R-Square  (predict)= 95.03% | | | R-Square (adjust) = 99.13% | S= 0.760 | |  |  |
|  |  | | |  |  | |  |  |

1. **Recovered lignin**

**Table S2.** Recent researches on clean fractionation for maximum yields of extracted lignin

| **References** | **Raw material** | **Conditions** | **Solvent mixture** | **Recovered lignin (%)** |
| --- | --- | --- | --- | --- |
| Brudecki et al., (2013) | Switchgrass | 0.75% H_2_SO_4_,  136 °C, 40 min | MIBK:ethanol:H_2_O | 92% |
| Wen et al., (2013) | Bamboo | 0.21-0.93% H_2_SO_4_,  120 °C,10-50 min | Formic acid:acetic acid: water | 21.7% |
| Cheiwpanich et al., (2017) | Bagasse | 0.025 M H_2_SO_4_,159 °C, 50 min | Ethyl acetate:methanol:water | 59.94% |
| Nopparat et al., (2017) | Sugar bagasse | 5%H_3_PO_4_,  180 °C, 60 min | Ethyl acetate:methanol:water | 87% |

**Table S3.** The specific surface area and crystallinity index of native corn stover and solid residue under optimized condition from solvothermal fractionation process

| **Sample** | **Surface area (m^2^/g)** |
| --- | --- |
| Native corn stover | 2.21 |
| Solid residue | 7.32 |

**Table S4.** Assignment of main recovered lignin signals in the ^13^C−^1^H 2D HSQC NMR spectra (from **Fig. 6**)

| Label | δ_C_/δ_H_ | Assignments |
| --- | --- | --- |
| C_β_ | 53.01/3.48 | C_β_-H_β_ in phenylcoumaran substructures |
| B_β_ | 52.61/3.17 | C_β_-H_β_ in β-β (resinol) substructures (B) |
| –OCH_3_ | 55.77/3.73 | C-H in methoxyls |
| A_γ_ | 60.15/3.47 and 3.61 | C_γ_-H_γ_ in β-O-4 substructure (A) |
| F_γ_ | 61.63/4.17 | C_γ_-H_γ_ in *p*-hydroxycinnamyl alcohol |
| A_α_ | 71.88/4.86 | C_α_−H_α_ in β-O-4 substructure (A) |
| S_2,6_ | 103.50/6.66 | C_2,6_-H_2,6_ in syringyl units (S) |
| S´_2,6_ | 106.5/7.22 | C_2,6_-H_2,6_ in oxidized S units (S´) |
| G_2_ | 110.47/6.90 | C_2_-H_2_ in guaiacyl units (G) |
| *p*CA_β_/FA_β_ | 115.36/6.26 | C_α_−H_α_ in *p*-coumarate and ferulate |
| *p*CA_α_/FA _α_ | 115.36/6.26 | C_α_−H_α_ in *p*-coumarate and ferulate |
| *p*CA_3,5_ | 115.60/6.93 | C_3,5_−H_3,5_ in *p*-coumarate |
| G_5_ | 115.60/6.77 | C_5_-H_5_ in guaiacyl units (G) |
| G_6_ | 118.62/6.74 | C_6_-H_6_ in guaiacyl units (G) |
| FA_6_ | 122.80/7.13 | C_6_-H_6_ _6_ in ferulate |
| G´_6_ | 123.16/7.52 | C_6_-H_6_ in oxidized (Cα=O) G units (G′) |
| H_2,6_ | 128.70/7.19 and 126.96/7.27 | C_2,6_-H_2,6_ in *p*-hydroxyphenyl units (H) |
| *p*CA_2,6_ | 130.17/7.49 | C_2,6_−H_2,6_ in *p*-coumarate |

**Table S5.** Py-GCMS analysis results of the identified compounds for commercial kraft lignin and recovered lignin from solvothermal fractionation

| Label | Compound | Origin | Commercial kraft lignin | Recovered lignin  (This study) |
| --- | --- | --- | --- | --- |
| 1 | phenol | H | 3.3 | 3.7 |
| 2 | 2-methylphenol | H | 2.3 | 0.8 |
| 3 | 4-methylphenol | H | 1.9 | N.D |
| 4 | guaiacol | G | 32.4 | 10.2 |
| 5 | 2,6-dimethylphenol | H | 0.9 | N.D |
| 6 | 2,4-dimethylphenol | H | N.D | 0.3 |
| 7 | 4-vinylphenol | H/PCA | N.D | 2.5 |
| 8 | methylguaiacol | G | 2.3 | 3.9 |
| 9 | 3-methoxy-1,2-benzenediol | S | N.D | 3.4 |
| 10 | 3,6-dimethylphenol | H | 1.3 | N.D |
| 11 | 4-methylguaiacol | G | 3.2 | N.D |
| 12 | 5-methylguaiacol | G | 5.3 | N.D |
| 13 | 4-ethylguaiacol | G | 3.9 | 2.0 |
| 14 | 4-vinylguaiacol | G/FA | 7.4 | 14.8 |
| 15 | 3-methoxy-5-methylphenol | H | N.D | 0.3 |
| 16 | allylguaiacol | G | 1.6 | 2.3 |
| 17 | syringol | S | N.D | 11.3 |
| 18 | 3,4-dimethoxyphenol | H | N.D | 0.6 |
| 19 | vanillin | G | 9.7 | 3.6 |
| 20 | cis-isoeugenol | G | 3.6 | 4.6 |
| 21 | trans-isoeugenol | G | N.D | 1.4 |
| 22 | 4-methylsyringol | S | N.D | 3.6 |
| 23 | 4-propylguaiacol | G | 2.1 | N.D |
| 24 | 3,4-dimethoxybenzaldehyde | G | 1.0 | N.D |
| 25 | acetoisovanillone | G | 5.4 | N.D |
| 26 | guaiacylacetone | G | 3.7 | 0.8 |
| 27 | 4-allylsyringol | S | N.D | 1.7 |
| 28 | syringaldehyde | S | N.D | 2.3 |
| 29 | trans-4-propenylsyringol | S | N.D | 6.2 |
| 30 | acetosyringone | S | N.D | 3.8 |
| 31 | cis-coniferyl alcohol | G | 0.8 | N.D |
| 32 | trans-coniferyl alcohol | G | N.D | 4.1 |
| 33 | propiosyringone | S | N.D | 0.8 |
| 34 | guaiacyl propanol | G | 6.0 | N.D |
| 35 | 4-hydroxy-2-methoxycinnamaldehyde | G | 2.0 | N.D |
| 36 | n-alkanes/n-alkenes | FatAc | N.D | 10.9 |
|  |  | %S ^[a]^ | 0.0 | 37.2 |
|  |  | %G ^[a]^ | 90.4 | 53.5 |
|  |  | %H ^[a]^ | 9.6 | 9.3 |
|  |  | S/G ^[a]^ | 0.0 | 0.70 |
|  |  |  |  |  |
|  |  | %S ^[b]^ | 0.0 | 46.2 |
|  |  | %G ^[b]^ | 89.6 | 45.7 |
|  |  | %H ^[b]^ | 10.4 | 8.1 |
|  |  | %G ^[b]^ | 0.00 | 1.0 |

^[a]^ Calculated by using all the H: p-hydroxyphenyl units; G: guaiacyl units and S: syringyl units; -lignin derived products

^[b]^ Estimated by ignoring 4-vinylphenol (mostly arising from p-coumarates) and 4-vinylguaiacol (which also arises from ferulates), and the analogous 4-vinylsyringol.
